# Droplet Microfluidics with MALDI-MS Detection: The Effects of Oil Phases in GABA Analysis

**DOI:** 10.1021/acsmeasuresciau.1c00017

**Published:** 2021-08-24

**Authors:** Sara E. Bell, Insu Park, Stanislav S. Rubakhin, Rashid Bashir, Yurii Vlasov, Jonathan V. Sweedler

**Affiliations:** † Department of Chemistry, University of Illinois Urbana−Champaign, Urbana, Illinois 61801, United States; ‡ Beckman Institute for Advanced Science and Technology, University of Illinois Urbana−Champaign, Urbana, Illinois 61801, United States; § Holonyak Micro & Nanotechnology Laboratory, University of Illinois Urbana−Champaign, Urbana, Illinois 61801, United States; ∥ Department of Electrical and Computer Engineering, University of Illinois Urbana−Champaign, Urbana, Illinois 61801, United States; ⊥ Department of Bioengineering, University of Illinois at Urbana−Champaign, Urbana, Illinois 61801, United States

**Keywords:** droplet microfluidics, mass spectrometry, MALDI, picoliter, neurotransmitter, GABA, perfluorinated oil

## Abstract

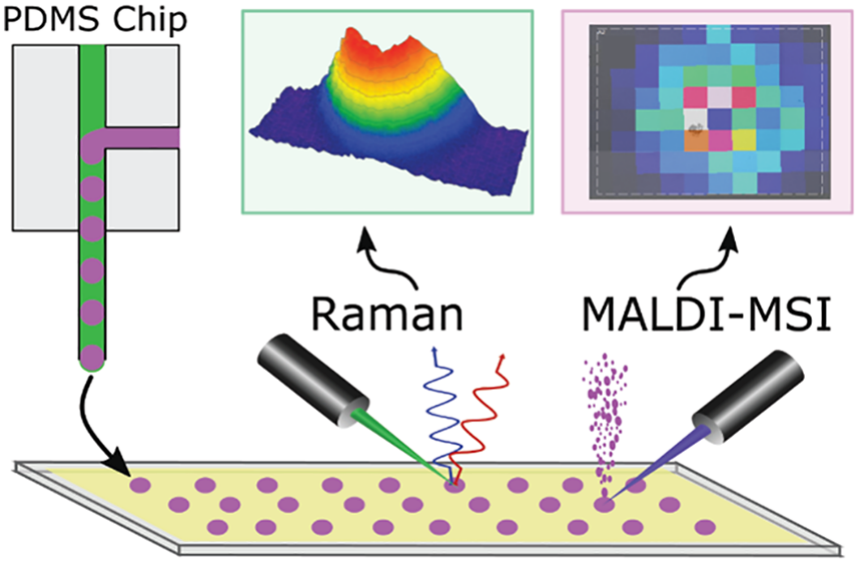

Microfluidic and mass spectrometry (MS) methods are widely used to sample and probe the chemical composition of biological systems to elucidate chemical correlates of their healthy and disease states. Though matrix-assisted laser desorption/ionization-mass spectrometry (MALDI)-MS has been hyphenated to droplet microfluidics for offline analyses, the effects of parameters related to droplet generation, such as the type of oil phase used, have been understudied. To characterize these effects, five different oil phases were tested in droplet microfluidics for producing samples for MALDI-MS analysis. Picoliter to nanoliter aqueous droplets containing 0.1 to 100 mM γ-aminobutyric acid (GABA) and inorganic salts were generated inside a polydimethylsiloxane microfluidic chip and deposited onto a conductive glass slide. Optical microscopy, Raman spectroscopy, and MALDI-mass spectrometry imaging (MSI) of the droplet samples and surrounding areas revealed patterns of solvent and oil evaporation and analyte deposition. Optical microscopy detected the presence of salt crystals in 50–100 μm diameter dried droplets, and Raman and MSI were used to correlate GABA signals to the visible droplet footprints. MALDI-MS analyses revealed that droplets prepared in the presence of octanol oil led to the poorest detectability of GABA, whereas the oil phases containing FC-40 provided the best detectability; GABA signal was localized to the footprint of 65 pL droplets with a limit of detection of 23 amol. The effect of the surfactant perfluorooctanol on analyte detection was also investigated.

## Introduction

Mass spectrometry (MS) is widely used for the analysis of chemical and biological systems owing to its highly sensitive, multiplexed, and untargeted nature.^1−3^ Similarly, the development of droplet microfluidic techniques has enabled high-throughput sample preparation and analysis of chemical and biological systems, in particular, the chemistry of the brain.^4−6^ Conventional brain microfluidic dialysis coupled to MS produces results on the time scale of minutes, whereas detection of small molecule neurochemicals by spectroscopy or electrochemistry has uncovered dynamic chemical signaling on the time scale of seconds.^7,8^ While fast, these latter techniques require analyte preselection and detection of fluorescence activity or electroactivity. In contrast, MS allows for multiplexed detection of neurochemicals on a fast time scale and is free from these limitations.^1,9,10^ To reach the second-scale time resolution, analytes may be packaged into individual picoliter-volume droplets, allowing preservation of chemical gradients obtained from dynamic biological systems by preventing analyte diffusion and biofouling.^11^ Analyte segmentation into a train of individual droplets requires the use of two immiscible phases, such as an oil and an aqueous phase (e.g., water or cerebral spinal fluid). Additionally, various surfactants are often used to stabilize the interface between the two phases,^12^ facilitating the generation, transport, and storage of droplets. Transfer of droplets to separate storage microarrays^13^ allows sample generation and MS analysis to be decoupled, increasing the choice of detection methods.

Many MS techniques^14−16^ can be utilized to measure droplet contents, including electrospray ionization (ESI),^5,17−19^ inductively coupled plasma (ICP),^20−22^ or matrix-assisted laser desorption/ionization (MALDI).^23−29^ Carrier oils and surfactants have been shown to interfere with spray stability and ion detection in ESI-MS,^30^ where droplet production and analysis are not always decoupled. However, when coupled to MALDI-MS, the influence of commonly used carrier oils and surfactants has not been well evaluated.

Previous studies of MALDI-MS detection of larger molecules, such as peptides and proteins,^26,27^ have not reported any adverse effects caused by the most commonly used fluorinated oils and surfactants; no oil residue has been observed on the sample substrate nor the presence of oil-related or surfactant-related mass spectral peaks. Though oils and surfactants in MALDI samples may not influence the detection of large molecules, their effect on the detectability of small molecules (in the range of most classical neurotransmitters) has not been well studied. Most of the recent reports on droplet-assisted MALDI sample preparation utilize electrowetting on dielectric devices that are capable of forming and manipulating aqueous droplets in ambient air without the presence of oils.^31−36^ Though Pereira et al.^29^ documented adverse effects of the perfluorinated oil FC-40 on MALDI matrix crystallization, a comparative study of different oils and surfactant-related effects on MALDI-MS detection of small molecule neurochemicals (<500 Da) in droplets is lacking.

Typically, microfluidic-generated droplets with volumes over 100 pL, often a few nanoliters, result in successful MS analysis of peptides and proteins. In a few cases, droplet volumes less than 100 pL have successfully been analyzed by ESI-MS^5^ and ICP-MS.^22^ For MALDI-MS analysis, the reduction of sample volumes to the low picoliter range has been shown by the application of continuous flow microfluidics with integrated microdispensers.^37−39^ However, development of droplet microfluidics sample preparation approaches in picoliter volumes requires additional characterization of the influence of the oil phase on different parameters of sampling and analyte detection.

To determine the effects of oils on sample preparation for MALDI-MS analysis, we tested a series of carrier oil phases for production of aqueous droplets containing γ-aminobutyric acid (GABA) in artificial cerebrospinal fluid (aCSF). First, we used optical microscopy, Raman spectroscopy, and mass spectrometry imaging (MSI) to validate analyte-droplet footprint colocalization on the nonpatterned surface of an indium–tin oxide (ITO) coated glass slide. This was done in contrast to previous studies utilizing a variety of alternating hydrophobic and hydrophilic patterns on MALDI substrates to effectively capture droplets and repel oil.^20,23,24,26−28,40−42^ By using a nonpatterned ITO slide, a substrate commonly used in MALDI-MS analysis, we were able to probe surface-oil-GABA interactions and characterize the dried droplet morphologies. Additionally, droplets produced using different oils were compared for GABA localization and detectability using MALDI-MS. Ultimately, we achieved a 23 amol limit of detection (LOD) for GABA in droplets produced in the oil phase FC-40: perfluorooctanol (PFO) (10:1 v/v). The work described here allowed generation and measurement of 65 pL droplets, which may become enabling for smaller diameter sampling systems such as push–pull capillary sampling of the brain.^17,19,43^ Furthermore, this work opens new perspectives for application of droplet microfluidics hyphenated with MALDI-MS to other sampling and measurement fields.

## Experimental Section

### Chemicals

aCSF was purchased from Tocris Bioscience (Bristol, UK). Fluorinert FC-40, PFO, perfluorodecalin (PFD), 1-octanol, GABA, and α-cyano-4-hydroxycinnamic acid (CHCA) were purchased from Sigma-Aldrich (St. Louis, MO). All chemicals were used without further purification.

### Polydimethylsiloxane (PDMS) Microfluidic Chip

For stable generation of droplets, PDMS chips, each with an integrated T-junction, were designed and fabricated with the channel width and depth varied from 10 to 50 μm and 50 to 100 μm, respectively. PDMS and curing agent (10:1 mixing ratio) were poured on the master wafer and cured for 2 h at 60 °C. Master PDMS was bonded to a flat PDMS plate with an activated hydrophilic surface produced by 2 min exposure to low-pressure oxygen plasma and baking overnight at 50 °C. Once fabricated, the microfluidic channels in the PDMS chip were made hydrophobic by application of Aquapel (Aquapel Glass Treatment, Cranberry Twp, PA, USA) for 5 min and then rinsed with isopropyl alcohol (IPA). The inlet and outlet of the PDMS chip were designed for fitting of two fused silica glass capillaries to avoid solution leakage. Two fused silica glass capillaries (150 μm outer diameter/50 μm inner diameter) were cut to approximately 3 cm and inserted into the outlet and inlet of the chip. Once fabricated and plumbed, the chip was again treated with Aquapel for 5 min and rinsed with IPA. Lastly, the oil used for the continuous phase was flushed through the channels of the chip helping to remove IPA residue and air bubbles. A new PDMS chip was used for each oil phase to avoid cross-contamination.

### Droplet Production and Deposition on MALDI Substrates

To simulate the conditions of biological samples, droplets were generated from aCSF containing differing concentrations of GABA. Injection of the dispersed, aqueous phase into an integrated T-junction with a constant supply of oil-based continuous phase resulted in the generation of aqueous droplets with volumes between 65 pL and 1000 pL at 2 to 10 Hz frequency (Figure S1). The dispersed aqueous phase contained 100 mM or 100 μM GABA in aCSF. The following oils and oil mixtures were evaluated as the continuous oil phase: (1) 1-octanol; (2) a fluorocarbon PFD (C10F18) supplemented with surfactant PFO (10:1 ratio, v/v); (3) a fluorocarbon oil Fluorinert FC-40 supplemented with surfactant PFO (10:1 ratio, v/v); (4) PFO; and (5) Fluorinert FC-40 (Table S1). Two syringe pumps (Pump 11-Pico Plus Elite, Harvard Apparatus, Holliston, MA), directly connected with the inlet fused silica capillaries through 100 μL syringes and connectors, controlled the flow rates of the dispersed and continuous phases. The flow rate ratio between the dispersed and continuous phases was adjusted from 0.05:10 to 5:10 (nL/min), depending on the channel cross section and droplet volume required (Figure S1).

Droplets were transferred off-chip to an ITO-coated glass slide (Delta Technologies, Loveland, CO). This was accomplished by mechanical contact of the outlet fused silica capillary to the surface of the ITO slide. Positioning of the capillary was controlled manually using x–y–z micromanipulators. Droplets were deposited in relation to fiducial markers engraved into the slide by a diamond tip pen.

### Optical Microscopy

After droplet deposition, the ITO slide was mounted for optical microscopy imaging using a Mitutoyo FS70 inspection microscope (Mitutoyo, Kawasaki, Japan) at a 90° angle and a Dino-Lite digital microscope, model AM73515MZT (Dino-Lite, New Taipei, Taiwan); 45° and 0° angle projections were used to record the evaporation of water and oil phases. All time-lapsed images of the evaporation process were recorded using 10× and 20× objective lenses and cameras with 20 frames/s capture rates. Each droplet evaporation area was analyzed using the time-lapsed images and an in-house code,^44^ created using MATLAB (Mathworks, Natick, MA) and based on a particle tracking algorithm.

After evaporation, the entire ITO slide surface was recorded by whole slide bright-field microscopy using an Axio Imager M2 (Carl Zeiss, Jena, Germany) in order to visualize droplet locations with respect to fiducial marks. Images were acquired with a 10× objective and tiled to cover the whole slide surface. Images were stitched and exported as TIFF files using Zen 2 (Carl Zeiss, Blue edition) software. These images were further used to guide MALDI-MSI. Individual droplets were imaged for further characterization of morphology both before and after MALDI matrix application using an Axiovert 25 inverted microscope (Carl Zeiss).

### Droplet Evaporation Analysis

The in-house MATLAB code^44^ was used to analyze the area changes and particle trajectories of each droplet and surrounding oil phase during evaporation. First, the two regions of interest corresponding to the oil and droplet phases were manually selected for separate analysis. Next, adaptive histogram equalization was applied to the raw images to enhance the contrast between the droplet and oil phases. Additionally, a pixel-wise adaptive low-pass Wiener filter was used to reduce noise. After filtering, threshold values distinguishing the droplet and oil phases from the background were defined to create a binary mask, converting the images to binary format. Using the binary mask, center coordinates and radii were determined in all regions of interest and at each time point during evaporation.

### MALDI Matrix Application

The MALDI matrix CHCA was applied by sublimation using a lab-constructed, glass sublimation chamber.^45,46^ An ITO slide was attached to the bottom of a coldfinger using conductive copper tape. A shallow foil boat, 50 mm × 5 mm, containing CHCA was attached to the bottom of the chamber by conductive copper tape. The coldfinger was inserted into the chamber and sealed with an O-ring. A mechanical pump was used to establish vacuum for 5 min. Then ice was added to the coldfinger and the slide allowed to cool for 5 min. A variable power supply (120 V at 55%) and heating mantle were used to heat the chamber for 16 min to sublime the CHCA. The chamber was removed from the heating mantle and allowed to cool for 5 min before vacuum was released and the slide removed.

### MALDI-TOF-MS

Droplets of different volumes (65–1000 pL) containing known concentrations of GABA ([M + H]^+^ ion signal at *m*/*z* 104.07) were profiled with MS. Mass spectra were acquired on an ultrafleXtreme MALDI TOF/TOF mass spectrometer with a frequency tripled Nd:YAG solid-state laser (Bruker Corp., Billerica, MA). FlexImaging (Bruker Corp.) was used to profile each droplet and a surrounding area of approximately 1 × 1 mm^2^ with a spatial resolution of either 100 × 100 μm^2^ or 50 × 50 μm^2^. The “Ultra” setting produced an ∼100 μm diameter laser beam footprint. Each pixel of the resulting molecular images corresponds to data collected by 100 laser shots.

### MALDI-MS Data Analysis

FlexImaging and ClinProTools software (Bruker Corp.) were used to visualize ion intensity distributions and extracted total ion count (TIC) normalized intensities for the *m*/*z* 104.07 ion, respectively. Principal component analysis (PCA) of data on analyte and matrix species observed in samples prepared using all studied conditions was carried out by ClinProTools. The TIC normalized intensities for *m*/*z* 104.07 were thresholded to determine the total GABA signal per droplet. First, the maximum intensity of the signal at *m*/*z* 104.07 detected in the blank area (corresponding to chemical noise and possible isobaric ions formed from MALDI matrix and/or the ITO-coated glass surface) was used as a threshold for the *m*/*z* 104.07 signal observed at each pixel in the MS images of droplet areas. Any signals with intensities below this threshold were excluded; all signal intensities above the threshold were summed to yield the total GABA signal intensity per droplet area. This thresholding technique is used for all cumulative MSI data and for construction of three-dimensional (3-D) heat maps of MS imaged areas in MATLAB.

### Raman Spectroscopy

Raman spectroscopy was performed using a Nanophoton Raman 11 laser confocal microscope (Nanophoton, Osaka, Japan) with an excitation wavelength of 532 nm. The excitation power was set at 20 and 50 mW for point spectra and mapping, respectively, with a 2 s exposure time and 5× averaging for both modes. For Raman spectroscopy mapping, the defined region of interest was 20 by 70 μm^2^ in the *x*–*y* plane with 1 pixel/μm resolution. The objective lens used was 50×, and the grating was 600 gr/mm. The wavenumber range covered was 400 to 2900 cm^–1^. The wavenumber shift compensation was −7.89 cm^–1^ after calibration using a standard, low-pressure neon lamp.

## Results and Discussion

### Droplet Morphology on MALDI Substrates

**Figure 1:**
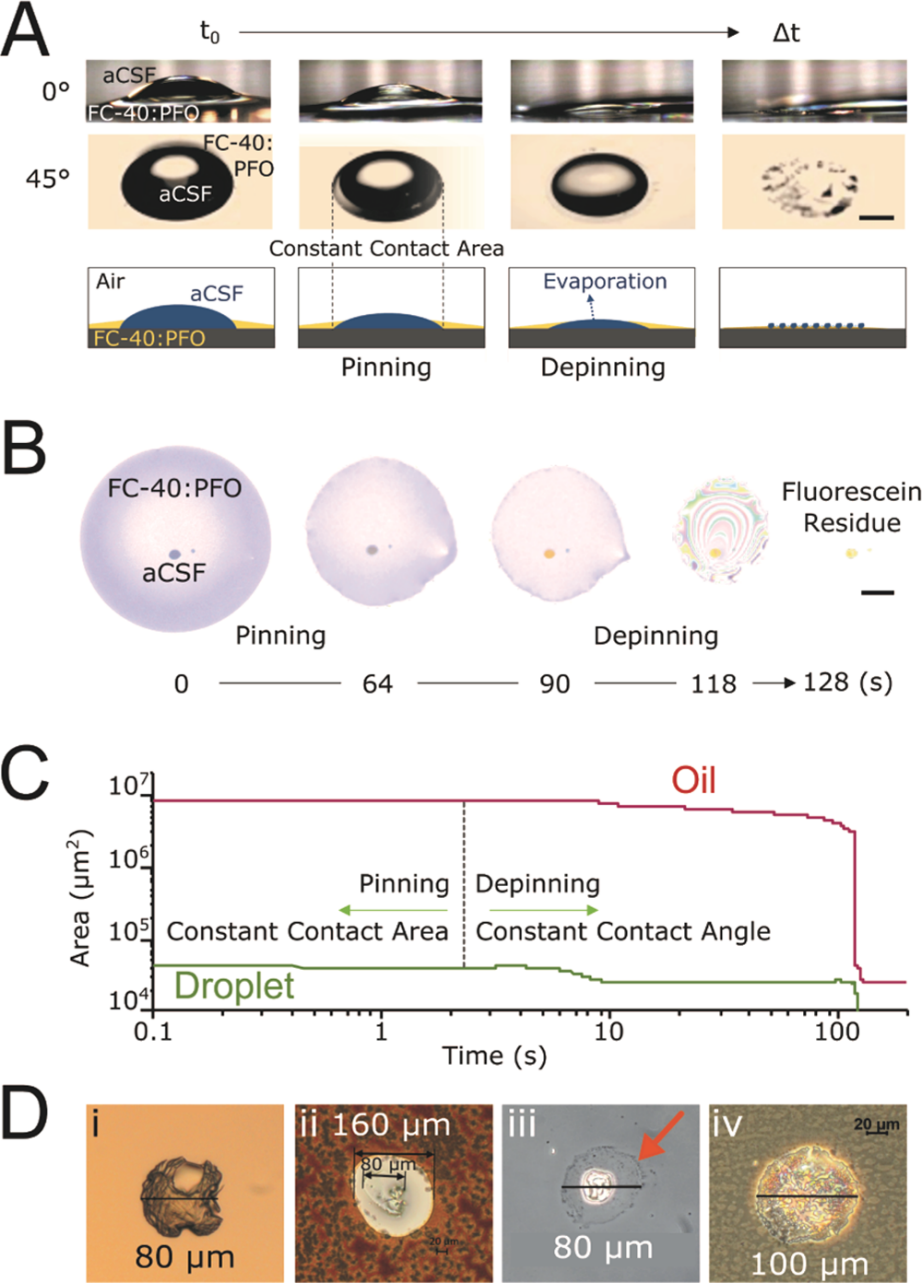
Dynamics of droplet drying and morphology on an ITO glass slide. Evaporation of picoliter sessile droplets and surrounding oil are responsible for size reduction of visible structures. (A) Time-lapsed microscope images of 500 nL aqueous droplet and FC40:PFO oil phase at a 0° and 45° angle projections. Scale bar, 500 μm. (B) Time-lapsed images of evaporation of 65 pL droplet containing 100 μM fluorescein and 100 μM GABA surrounded by oil phase. Scale bar, 150 μm. (C) Time course of pinning and depinning phases of droplet on ITO substrate. Droplet and oil are completely dried in 10 and 100 s, respectively. (D) Dried cores of droplets produced in FC-40:PFO for 1000 pL volumes (i) before matrix and (ii) after matrix application; and for 65 pL volumes (iii) before matrix and (iv) after matrix. The oil residue halo surrounding the droplet core is visible in (iii) (marked by an orange arrow).

To understand how aqueous droplets and the surrounding oil interact with the ITO glass substrate surface, we performed a series of optical microscopy studies to visualize the droplets. Once single droplets were deposited onto the ITO surface, two cameras were used to track the evaporation of both aqueous and oil phases in 45° and 0° angle projections, respectively (Figure 1A). Upon single droplet deposition, a large area was covered by both the oil phase and aqueous phase (e.g., 800 μm diameter for a 65 pL droplet). Due to the hydrophobic nature of the ITO surface, the contact angle of the aqueous picoliter droplet was larger than that of the oil, protruding above the surface of the oil, and started to evaporate almost immediately after deposition. To analyze the water and oil evaporation rates, fluorescein was added into a series of 65 pL aCSF droplets to clearly visualize changes in droplet morphology and formation of the dried droplet deposit (Figure 1B). Particle tracking analysis^44^ revealed two stages of size reduction in the deposited droplets, pinning and depinning,^47^ characterized by constant contact area and constant contact angle, respectively (Figure 1C). During this process, the aqueous droplet held its position on the ITO surface, revealing minor in-plane motion (Figure S2). Due to the lack of droplet translation during evaporation, we expected the analyte to be localized to the dried droplet deposit visible after evaporation was complete.

After evaporation, the morphological features of droplet volumes ranging from 65 to 1000 pL were characterized both before and after MALDI matrix application (Figure 1D). For all volumes, a 50 to 100 μm diameter deposit, referred to as the droplet core, remained surrounded by a faint outline, referred to as the droplet halo (orange arrow in Figure 1D(iii)). For 1000 pL droplets, inorganic salts present in the aCSF crystallized to develop the droplet core, which did not integrate well with the MALDI matrix (Figure 1D(i) and (ii)). The lack of matrix–analyte contact and cocrystallization resulted in poor ionization of GABA at this position (Figure S3). GABA signals acquired from the salt crystal positions were 2 orders of magnitude lower in intensity compared to signals observed at the adjacent pixels. This phenomenon was observed for volumes of 400 pL or greater. For droplet volumes below 400 pL, images show only partial or no internal crystallization within the droplet core (Figure 1D(iii)). In addition, the less-abundant presence of crystals allowed for better integration of MALDI matrix with the droplet core (Figure 1D(iv)). In both cases, droplet cores that remained after evaporation were typically smaller than the 100 μm diameter laser beam used for MALDI-MSI. To determine both GABA localization and detectability, the observed morphological features (core and halo) can be further correlated with molecular maps of the distribution of different compounds determined by Raman spectroscopy and MSI. Based on these observations, we anticipated that the small volumes would produce better MS signal and both Raman and MS results to show GABA localized to the droplet core.

### GABA Localization in Dried Droplets Determined by Raman Spectroscopy

Application of MALDI matrix solutions onto samples by nebulizing sprayers or airbrushing can induce analyte delocalization due to diffusion and/or mechanical forces.^48−50^ To minimize this undesirable effect, solvent-free MALDI matrix application by sublimation was used for all droplet-based samples. Previously, this approach demonstrated the least amount of delocalization of analytes for enhanced MS image quality.^46^ However, humidity^50^ and water condensation on the ITO slide surface can cause analyte delocalization similar to that caused by solvent-based MALDI matrix application approaches. Unfortunately, the presence of inorganic salts may induce signal suppression as well as a nonhomogeneous matrix coating, resulting in varying degrees of matrix-analyte cocrystallization. MALDI-MSI results may be strongly affected by these phenomena due to a mixture of both “hot spots” with enhanced signal and areas of diminished signal. Therefore, it is important to examine the effects of MALDI matrix application on analyte detection and localization in samples by using an approach orthogonal to MS.

**Figure 2:**
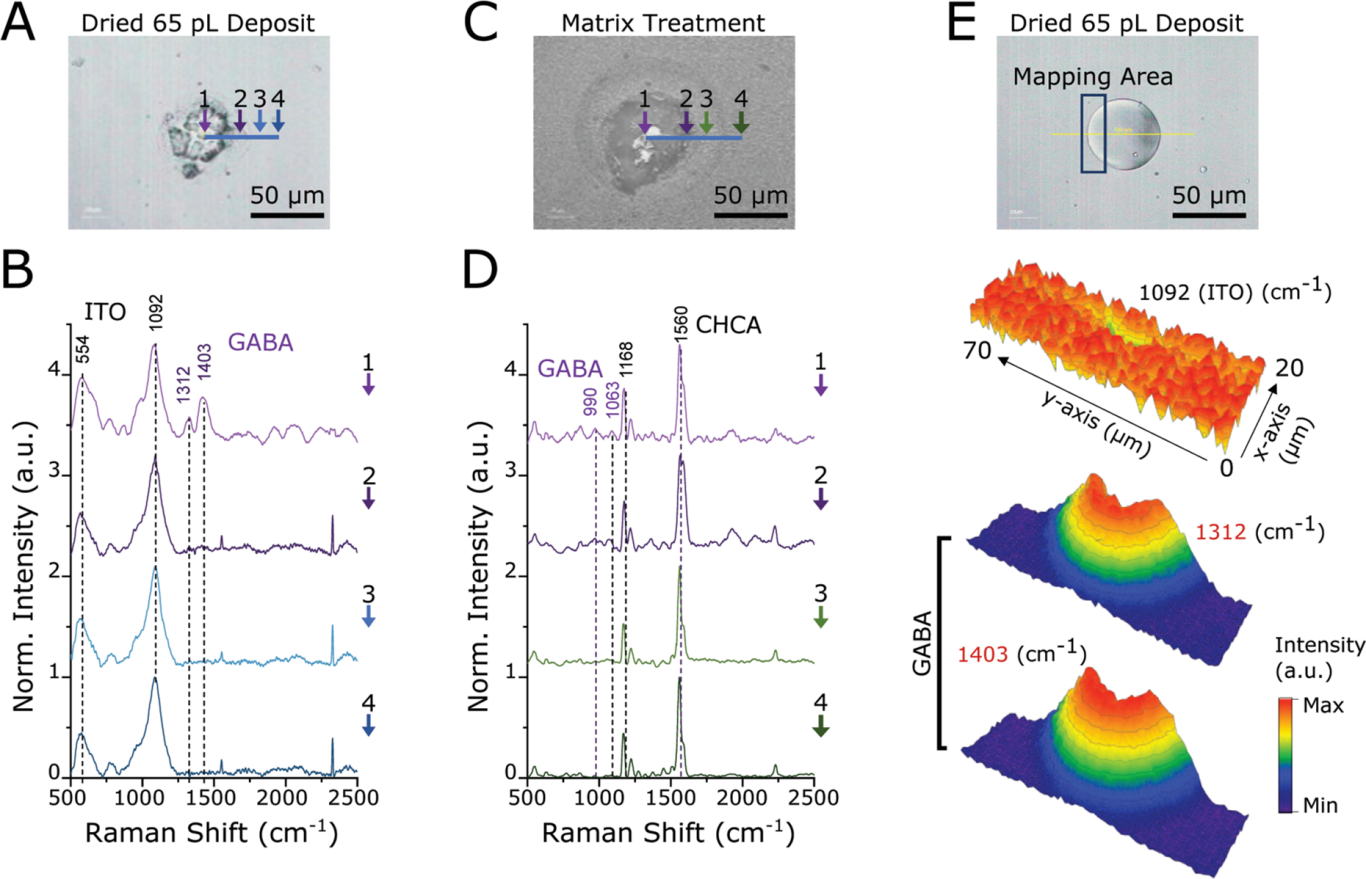
Raman spectroscopy characterization of dried droplets produced in FC-40:PFO. (A) Upright microscope image of the dried droplet after evaporation. Crystals formed mostly by inorganic salts present in aCSF are visible at the point marked 1; point 2 marks the outer edge of the droplet core; point 3 marks the droplet’s halo; point 4 marks the area outside of the droplet and represents the controls/blanks. Points 2–4, respectively, occur at distances 25, 38, and 50 μm from point 1. (B) Raman spectra measured at locations corresponding to points 1–4. (C) Upright microscope image of the dried droplet after MALDI matrix application by sublimation. Points 1–4 correspond to the same droplet areas as in (A); points 2–4, respectively, occur at distances 28, 42, and 65 μm from point 1. (D) Raman spectra measured at the corresponding droplet locations taken after MALDI matrix application. (E) Raman mapping of the droplet area before matrix deposition at frequencies of ITO peak (1092 cm^–1^) and GABA peaks (1312 and 1403 cm^–1^).

To this end, Raman spectroscopy was utilized to determine analyte location with respect to the dried droplet and surrounding areas (Figure 2). These measurements also allowed further evaluation of the influence of droplet deposition and solvent evaporation stages as well as provided higher spatial resolution than MALDI-MSI. For these experiments, 100 mM GABA, 65 pL aCSF droplets produced in FC-40:PFO were further investigated. As seen in our optical studies (Figure 1), the diameter of the dried droplet core is ∼50 μm in the *x*–*y* plane (Figure 2A), with a surrounding halo visible in approximately 50% of droplets. Once matrix was applied, the outer halo surrounding the droplet cores, with a diameter of ∼100 μm, was more consistently visible (Figure 2C). Within the halo region, the CHCA MALDI matrix density was reduced, with a lack of matrix around the perimeter (Figure 2C). A similar halo region and change in matrix density was seen for a majority of droplets produced in all oils. Since CHCA is 25× more polar than FC-40, the decrease in matrix density suggests increased hydrophobicity of the substrate from incomplete oil phase evaporation or substrate modification by the oil phase within the halo region.

Raman spectroscopy was performed on the same droplets before and after MALDI matrix application for comparison of GABA-related signals. Prominent signals related to the ITO substrate were detected at 554 and 1092 cm^–1^; signals specific to the CHCA matrix were observed at 1168 and 1560 cm^–1^. The GABA signals at 1063 cm^–1^ (CH_2_ bending), 1312 cm^–1^ (CH_2_ bending), and 1403 cm^–1^ (COO^–^ symmetric stretch) were detected at the center (point 1) and edge (point 2) of the crystalline droplet core and were not detectable just 10 μm outside (point 3), both before and after matrix deposition (Figure 2B and D). Raman mapping was then performed on droplets across an area 20 × 70 μm^2^ in the *x*–*y* plane. Intensity profiles for both the prominent ITO peak and GABA peaks (Figure 2E) indicate that the concentration of GABA outside of the droplet core fell below the Raman spectroscopy LOD of about 1 nmol (see Figure S4). This suggests that the visible halo deposit is not formed by dislocation of droplet contents during the matrix application process but can be attributed to oil residue.

### GABA Signal Intensity Is Affected by the Oil Phase

To accurately perform GABA spatial mapping with MSI, we first had to determine if the oil phases affect the detection of GABA in single droplets. Our study focuses on the use of fluorinated oils for droplet production because most organic compounds are insoluble and do not partition to these phases.^12^ Octanol was chosen as a comparison due to its nonfluorinated nature and lower vapor pressure. In this way, we were able to compare the effects of fluorination, vapor pressure/drying time, and surfactant-character on MS detection of GABA.

Between five and eight droplets produced in oil phases FC-40:PFO, PFD:PFO, PFO, or octanol were investigated with MALDI-MSI. For each droplet, the total area imaged was on average 1 × 1 mm^2^ in order to include all pixels corresponding to the area covered by the aqueous droplet and surrounding oil during deposition, as well as pixels representative of the blank corresponding to the MALDI matrix. Consistently, the GABA [M + H]^+^ ion images showed the most intense signal for *m*/*z* 104.07 at the pixel corresponding to the droplet core and a few adjacent pixels. Additional [M + H]^+^ signal of 10× lower intensity was also detected in the surrounding area at an ∼400 μm radius. This area of low intensity GABA signal corresponded to the area covered by oil during deposition and the droplet’s halo. Other ions were also detected, localized to the same pixels as the GABA [M + H]^+^ ion, including a sodiated CHCA matrix [M + Na]^+^ at *m*/*z* 212.07, a putative GABA dimer [2 M + H]^+^ at *m*/*z* 208.14, and an unidentified signal at *m*/*z* 656.03.

**Figure 3:**
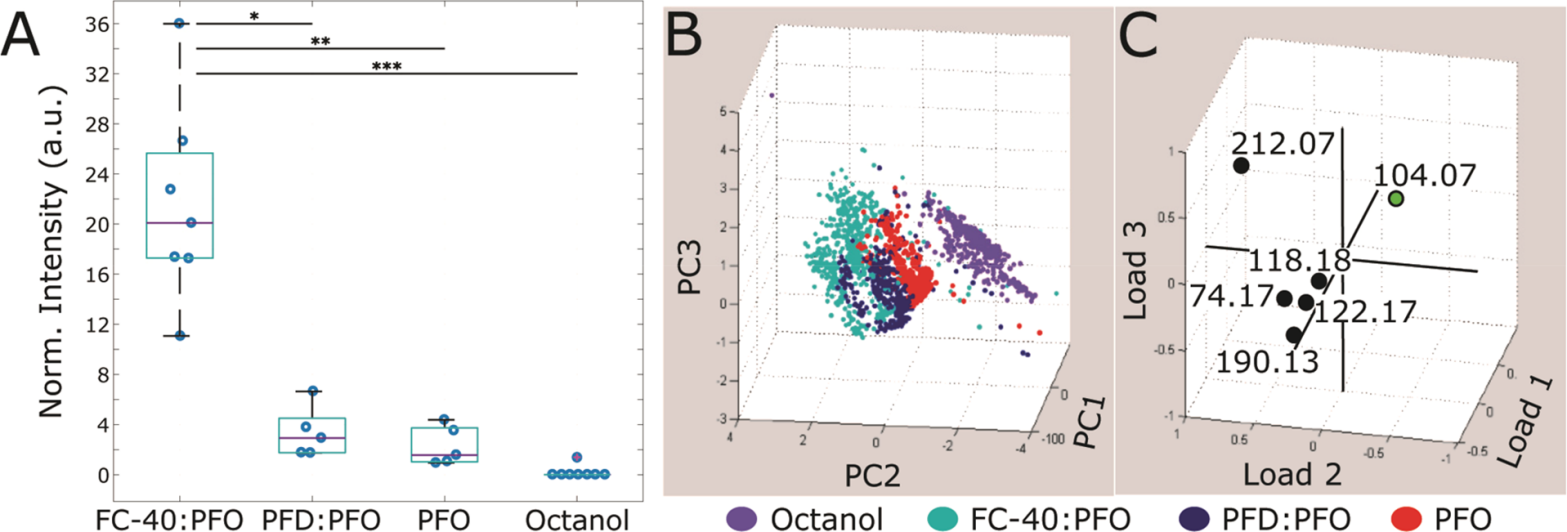
Effect of oil on summed and individual pixel intensities of selected mass spectral features. (A) Summed GABA signal intensity per droplet is plotted for each oil. Each blue circle represents the summed, thresholded GABA signal intensity for each singular dried droplet. Droplets produced in FC-40 and octanol are 65 pL, and droplets produced in PFD and PFO are 200 pL. The summed intensity is normalized to volume to account for this difference. Each oil had between five and eight individual dried droplets examined. Significantly higher signal intensity is seen for droplets generated using FC-40 with 10% PFO where *p = 0.04, **p = 0.003, ***p = 0.00002 (by one-way ANOVA). In the case of droplets produced in PFD with 10% PFO or PFO, summed signal intensities are not significantly higher (*p* > 0.05) compared to octanol-produced droplets. (B, C) PCA plots for the data collected from samples prepared using the four oil phases, where (B) shows single pixel principal component scores for each oil phase type and (C) shows loading plot with GABA [M + H]^+^*m*/*z* 104.07 ion and MALDI matrix-related signals at *m*/*z* 74.17, 118.18, 122.17, 190.13, and 212.07.

All signals associated with droplet contents were detected across multiple pixels. To evaluate MALDI detection capabilities with the different oil phases, the total GABA signal detected for each imaged area was summed using the thresholding technique described above. After determination of total signal for each droplet, we found that GABA total signal intensity was significantly higher for droplets prepared using the FC-40:PFO mixture than for droplets made using the other three oils (Figure 3A). Most strikingly, in the presence of octanol the GABA signal intensity was lowest and often below the background formed by chemical noise and other ions, likely related to the MALDI matrix. To further understand these differences in GABA detectability, PCA was used to compare the MALDI matrix ion profile of all droplet samples (Figure 3B, C). This analysis revealed different ionization profiles for perfluorinated oils vs unfluorinated. All perfluorinated oil-related samples showed the expected ion profile for CHCA matrix, whereas octanol-related samples produced fewer MALDI matrix-related signals, along with lower GABA signal intensities (Figure 3A). These differences in not only GABA detection but also in MALDI matrix detection suggest that residual octanol may interfere with the ionization process. This also further supports conclusions from our microscopy and Raman studies that the observed droplet halo may be formed by oil residue or substrate modification by the oil.

When comparing the mass spectra of droplet samples prepared with both FC-40 and octanol to the blank MALDI matrix spectra (Figure S5), multiple signals are colocalized with the droplet area that are not attributable to the MALDI matrix nor to GABA. However, none could be identified as FC-40 or octanol and could be due to the known poor ionizability of the oils. Alternatively, the colocalized signals may represent contaminants that are present in the oil phase. Despite the possible presence of contaminants in both oils, the FC-40:PFO oil phase resulted in the least amount of interference with GABA detection. Accordingly, we determined that FC-40:PFO was the best performing of the four oil mixtures for MALDI-MSI.

### GABA Signal Localization in Dried Droplets Determined by MSI

Spreading of analyte on the surface of the MALDI substrate during droplet deposition and MALDI matrix application is another factor influencing the intensity of analyte signals and, therefore, one of the important factors limiting the sensitivity of detection. If GABA is spread and delocalized, the moles of GABA per unit area will decrease and require a lower LOD for detection; thus, delocalization of GABA from the droplet core would be detrimental to the detection capabilities of our system. Our Raman spectroscopy studies indicate that the GABA concentration outside of the droplet core is below the 1 nmol LOD. To further quantify GABA localization, we performed MSI on droplet cores, halos, and surrounding areas.

**Figure 4:**
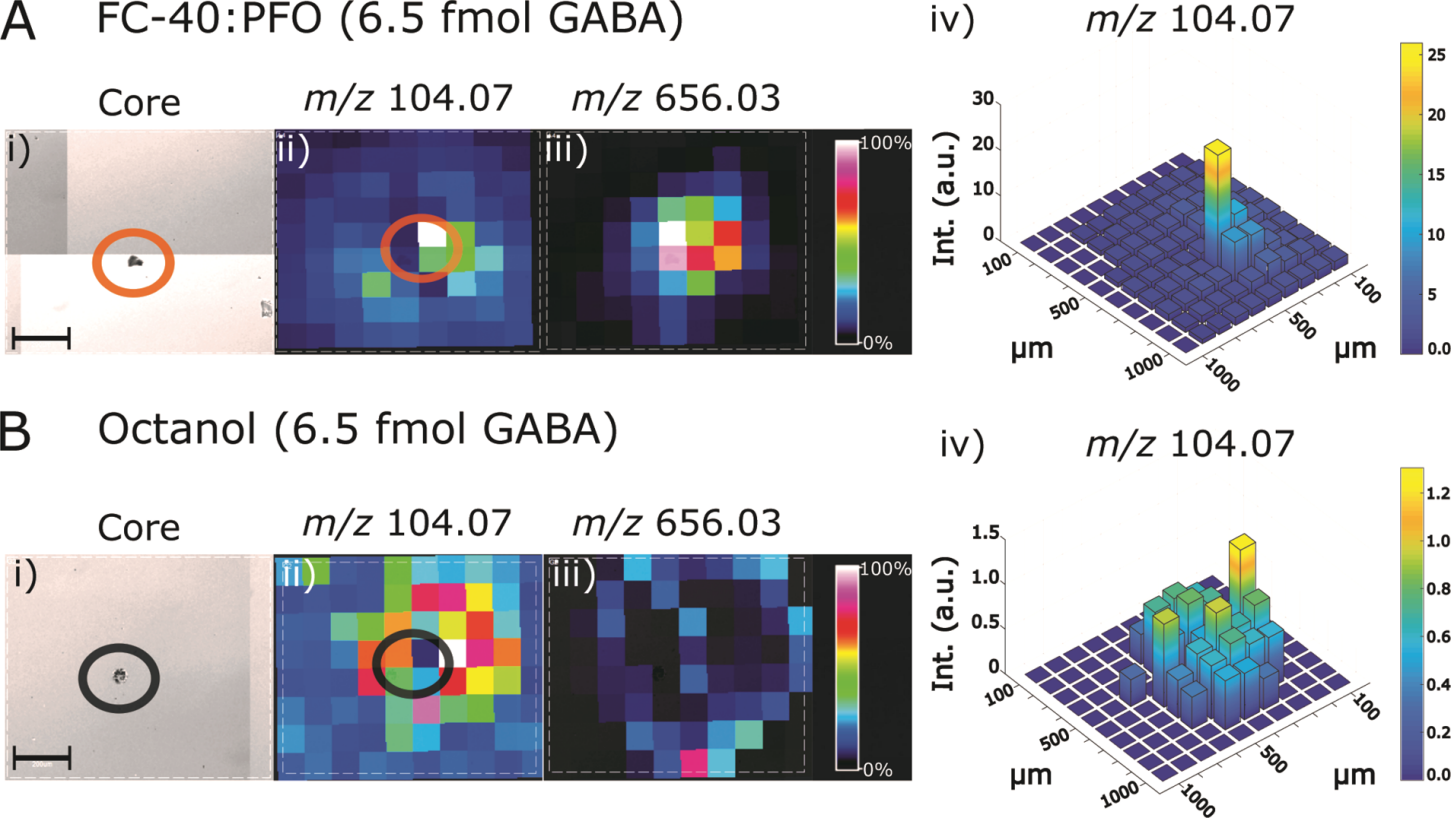
MALDI-MS imaging of dried droplets. (A) 65 pL droplets containing 6.5 fmol of GABA produced in the FC-40:PFO oil phase. Panel (i) shows an optical image of the dried droplet core outlined by an orange circle. Panel (ii) shows the MS ion image for *m*/*z* 104.07 corresponding to the GABA [M + H]^+^ ion, where the orange circle denotes the same area as shown in (i); the core is centered between 4 pixels in the MS image. Panel (iii) shows the MS ion image for colocalized signal at *m*/*z* 656.03. Panel (iv) shows a 3-D heat map of *m*/*z* 104.07 ion image to quantify the extent of signal spreading by calculation of the fwhm of the physical distribution of GABA. For FC-40:PFO, the fwhm is 100 μm. (B) 65 pL droplets containing 6.5 fmol of GABA produced in the octanol oil phase. Panel (i) optical image of the dried droplet core outlined by a black circle. Panel (ii) shows the MS ion image for *m*/*z* 104.07 corresponding to the GABA [M + H]^+^ ion where the black circle denotes the same area as shown in (i), the core is centered between 4 pixels in the MS image. Panel (iii) shows the MS ion image for colocalized signal at *m*/*z* 656.03. Panel (iv) shows a 3-D heat map of *m*/*z* 104.07 ion image where the fwhm of GABA distribution is 500 μm. Scale bars in panels (i) are 200 μm. Pixels are 100 μm in panels (ii)–(iv).

MSI was performed on 65–1000 pL aqueous droplets containing 6.5 fmol to 100 pmol of GABA produced in FC-40:PFO or octanol oil phases. For all droplets, the highest intensity signals of [M + H]^+^ at *m*/*z* 104.07 were detected in the droplet core (Figure 4A(ii) and B(ii)). Circular patterns of ∼10× lower intensity signal for *m*/*z* 104.07 were detected in the surrounding area that is occupied by the oil phase during deposition and the droplet’s halo. Typically, the signal for *m*/*z* 104.07 was delocalized to a diameter 3–4 times larger than the droplet core (e.g., ∼600 μm diameter *m*/*z* 104.07 ion images vs 50–100 μm droplet core). Additionally, unidentified, colocalized signals were detected across the same area as *m*/*z* 104.07 GABA signals (Figure 4A(iii) and B(iii)). This extent of delocalization of GABA and related signals was observed across different droplet volumes and therefore moles of GABA per droplet (Figure S6). Furthermore, for all droplet volumes produced in the FC-40:PFO and octanol oil phases, the theoretical droplet contact area on the ITO-glass slide (Figure S7) underestimated the ion image areas for *m*/*z* 104.07 signal by one to 2 orders of magnitude. Since the observed ion patterns were consistently larger than the droplet core, we hypothesized that the presence of surfactant-like molecules (PFO or octanol) may act as a permeable interface^51,52^ between GABA and the oil phase or the hydrophobic ITO-glass surface. However, even after removal of the surfactant, GABA ion images for droplets produced in FC-40 exhibited the same extent of delocalization (Figure S6). Unlike the observations from Raman imaging of droplets, the MS data suggests that GABA is not localized only to the droplet core. Furthermore, this analysis was not able to attribute the cause of the delocalization phenomenon observed in MSI to the oil phase properties or droplet volume.

To better understand the extent of delocalization, the pattern of GABA MSI signals was further quantified. To this end, the *m*/*z* 104.07 [M + H]^+^ ion images were thresholded using the blank, MALDI matrix signal at *m*/*z* 104.07. The full-width half-maximum (fwhm) was determined for the thresholded distribution of GABA signal in the *x*–*y* plane of the ion image (Figure 4). For droplets made in FC-40:PFO (Figure 4A(iv)), the fwhm of the [M + H]^+^ distribution was 100–200 μm in the *x*–*y* plane. This fwhm was also consistent for droplets of different volumes: 1000 vs 65 pL (Figure S6). Furthermore, the removal of the surfactant PFO in the FC-40 oil phase did not affect the fwhm of the intensity profile (Figure S6). Given that the focused laser spot diameter was 100 μm, the image pixel width was 100 μm, and droplet cores were ≤100 μm in diameter, the droplet core was imaged within a maximum of 2 × 2 pixels in the *x*–*y* plane, depending on alignment of the laser with the core. This analysis shows that the dimensions of the fwhm and the droplet core correspond to the same 2 × 2 pixels in the *x*–*y* plane. Furthermore, this fwhm of the ion distribution overlaps with both the droplet core seen in the optical images and the area of high intensity GABA signals observed by Raman spectroscopy. Therefore, in the FC-40:PFO-mediated samples, a majority of the GABA signals were localized to the droplet core.

In contrast, with octanol as the oil phase, the peak intensity decreased 16-fold while the fwhm increased 2.5-fold (Figure 4B(iv)). These results suggest that analyte spreading may be partially dependent on oil phase properties such as vapor pressure^53^ and surfactant character. The time scale of solute exchange from the aqueous to oil phase ranges from minutes to days depending on analyte identity.^51^ When using the FC-40:PFO oil phase, droplet generation, deposition, and drying occur within 2 min. In this case, we expect GABA exchange to be minimal in FC-40. Using octanol, the majority of the oil evaporates in ∼2 min; however, complete evaporation of oil residue occurs over ∼24 h, which may increase the chance for exchange. Overall, this shows that droplets produced in FC-40-based oils generate the highest intensity GABA signals localized to the ≤100 μm diameter droplet cores.

### Concentration Dependence of MALDI-MS Detection of GABA

**Figure 5:**
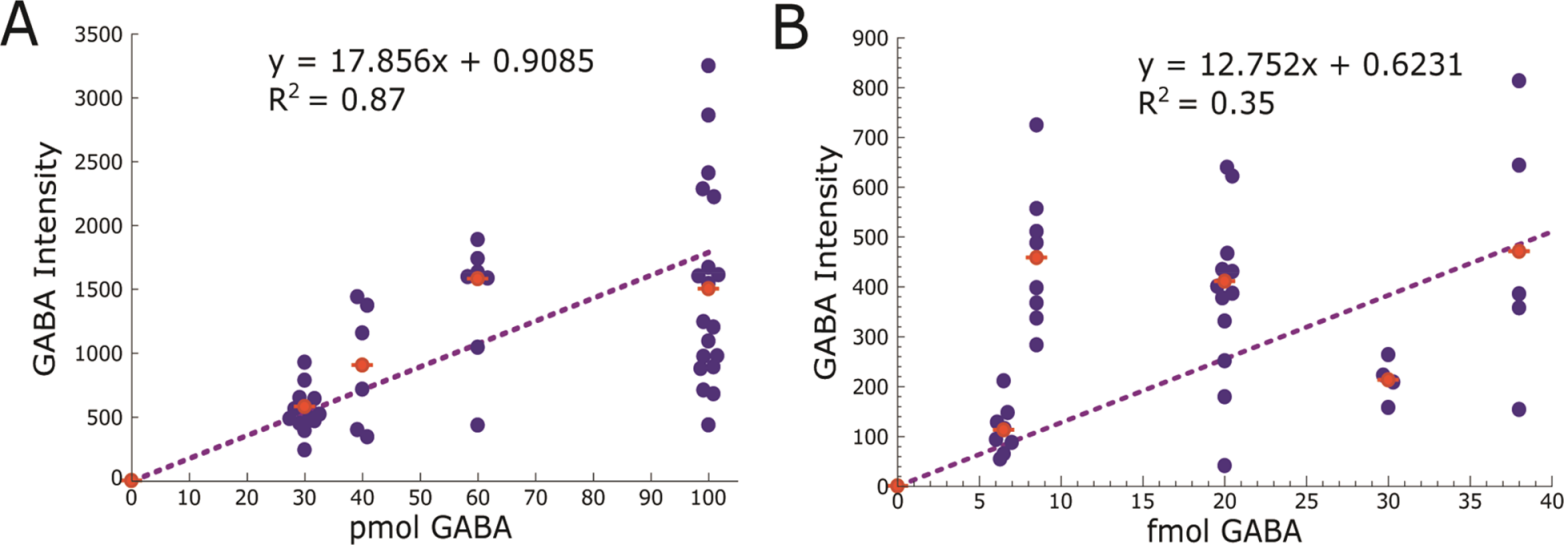
Dependence of GABA [M + H]^+^ ion signal intensity on amount of GABA per droplet. Each data point corresponds to the thresholded and summed intensity of GABA signal in single dried droplets. Droplets were produced in FC-40:PFO oil phase to create two concentration regimes: 100 mM and 100 μM. (A) 100 mM concentration regime for volumes 300 to 1000 pL corresponding to 30, 40, 60, and 100 pmol of GABA per droplet, respectively. Linear regression for the pmol range, *y* = 17.856*x* + 0.9085, *R*^2^ = 0.87; where the intercept is set at the mean blank signal. LOD is calculated by mean_blank_ + 3SD_blank_ and is equal to 122 fmol. (B) 100 μM concentration regime for volumes 65–380 pL corresponding to 6.5, 8.5, 20, 30, and 38 fmol of GABA per droplet, respectively. Linear regression for the fmol range, *y* = 12.752*x* + 0.6231, *R*^2^ = 0.35; where the intercept is set at the mean blank signal. LOD is calculated by mean_blank_ + 3SD_blank_ and is equal to 23 amol.

Since FC-40-based oil phases produced both the lowest analyte spreading and the best signal intensities, we evaluated the MALDI-MSI linearity and LOD using droplets produced in the FC-40:PFO oil phase (Figure 5). We studied two concentration regimes: 1) 100 mM GABA in droplet volumes from 300 pL to 1000 pL and 2) 100 μM GABA in droplet volumes 65 pL to 380 pL. These volumes and concentrations correspond to 30 to 100 pmol and 6.5 to 38 fmol of GABA for each regime, respectively. The dependence of signal on droplet volume, and therefore on moles of GABA deposited, for each concentration regime can be fitted to respective linear regressions. For the pmol range of GABA (Figure 5A), the calculated LOD is 122 fmol. This LOD is well above the target biological concentrations^19^ in sub-100 pL droplets taken from the brain. However, data points for the 1000 pL droplets (corresponding to 100 pmol) level off, possibly due to detector saturation^54^ at high concentrations or a reduction in analyte signal due to large salt deposits. Overall, these results in the large-volume regime illustrate the complex interaction between the droplet’s inorganic salts, MALDI matrix, and analyte with MS peak intensity. As expected, the high amount of salts in large volumes leads to completely crystallized droplet cores and visible artifacts in the MALDI matrix coating, leading to poor GABA ionization (Figure S3). Due to the salt interference with ionization, it is less favorable to produce volumes of 400 pL or greater when dealing with salty biological samples.

Our goal was to detect small amounts of GABA in sub-100 pL volumes that exhibit less extensive crystallization. Correspondingly, in the small volume range from 65 to 380 pL, we see less salt interference in GABA detection at the droplet core. The observed variability in small-volume performance may be attributed in part to the manually controlled hardware. Compared to automated deposition techniques,^23,26,27^ the use of manual deposition produces less repeatable droplet measurements. In response, future work will be focused on improving deposition through automated device handling. Results from these droplet samples containing 6.5–38 fmol of GABA were used for linear regression (Figure 5B), with the intercept set at the mean background signal for *m*/*z* 104.07. From the resulting trendline, the LOD is determined to be 23 amol of GABA.

These results indicate that the analysis of samples prepared using smaller droplets is favorable when working with salty solutions, since the total amount of salt per droplet decreases. Furthermore, removal of PFO surfactant when producing 65 pL droplets led to an increase in total ion intensity detected in droplets containing 65 pmol and 6.5 fmol of GABA (green data points in Figure S8). This increase is most prominent at 6.5 fmol of GABA, most likely due to a decrease in ion suppression that occurs in surfactant-mediated droplets when the amount of PFO molecules nears or surpasses the number of GABA molecules. Accordingly, we observed a 10-fold increase in average intensity in 65 pL, surfactant-free droplets containing 6.5 fmol of GABA. Since both lower volumes and surfactant-free droplets reduce ion suppression by the removal of interfering substances (salt and surfactant, respectively), the combination of these parameters improves the GABA signal. More work is needed to investigate possible improvement of the LOD with surfactant-free droplets. Overall, this work allowed us to develop a droplet production and MS analysis pipeline producing a 23 amol LOD for GABA.

## Conclusions

We performed an analytical evaluation of droplet microfluidic-assisted sample preparation parameters and determined their effects on MALDI-MSI detection of a classical neurotransmitter, GABA. Our results demonstrate the ability of droplet microfluidics coupled with MALDI-MS detection to analyze the contents of 65 pL droplets, the smallest volume measured with this approach so far. Additionally, we found that, at sub-400 pL volumes, the salt content of droplets likely has minimal influence on analyte detection, encouraging efforts to further reduce droplet size. FC-40 oil was shown to produce the highest localization of the GABA signal to the dried droplet core along with the highest intensity MS signals, allowing us to achieve a LOD of 23 amol for GABA detection. Furthermore, removal of the surfactant lead to a 10-fold increase in intensity of GABA signal; however, more studies are needed to determine any effect on LOD.

In summary, we have shown that analytical evaluation of the oil phase for droplet generation is important for MS method development. Our analysis of different experimental parameters allowed us to successfully hyphenate droplet microfluidic-assisted sample generation with off-line MALDI-MS to achieve attomole levels of detection, laying the groundwork for future analysis of neurochemical systems in low picoliter-volume droplets.

## Supporting Information Available

Droplet sample generation parameters; droplet volume control and deposition on MALDI substrate; trajectory analysis of evaporating droplet movement; representative mass spectra acquired at the crystalline droplet sample core and an adjacent pixel; characterization of Raman performance for MALDI-MSI samples; representative subtracted mass spectra acquired from samples prepared using droplets produced in FC-40 and octanol; comparison of MS imaging patterns of ion distribution across different droplet production parameters; calculation of sessile droplet areas deposited on ITO substrate; comparison of total GABA intensity for droplets produced in surfactant containing vs surfactant-free oil phases (PDF)
