# Supplementary material for: Droplet Microfluidics with MALDI-MS Detection: The Effects of Oil Phases in GABA Analysis
Source: ACS Meas Sci Au. 2021 Aug 24;1(3):147–56. doi: 10.1021/acsmeasuresciau.1c00017 (PMC8679089; doi:10.1021/acsmeasuresciau.1c00017)
Supplement: Supplementary file 1 — tg1c00017_si_001.pdf [file tg1c00017_si_001.pdf]

Supporting Information

Droplet Microfluidics with MALDI-MS Detection: The Effects of Oil Phases in GABA Analysis

Sara E. Bell[1,2#], Insu Park[3#], Stanislav S. Rubakhin[1,2], Rashid Bashir[2,3,4,5], Yurii Vlasov[2,3,4,5], Jonathan V. Sweedler[1,2,5,*]

[1]Department of Chemistry, University of Illinois Urbana−Champaign, Illinois 61801, USA

[2]Beckman Institute for Advanced Science and Technology, University of Illinois Urbana−Champaign, Illinois 61801, USA

[3]Nick Holonyak Jr. Micro and Nanotechnology Laboratory, University of Illinois Urbana–Champaign, Urbana, IL 61801, USA

[4]Department of Electrical and Computer Engineering, University of Illinois Urbana–Champaign, Urbana, IL 61801, USA

[5]Department of Bioengineering, University of Illinois at Urbana–Champaign, Urbana, IL 61801, USA

#SEB and IP are co-first authors with equal contribution to the work.

*Corresponding author: jsweedle@illinois.edu

Table of Contents

Table S1 .... S2
Figure S1 .... S3
Figure S2 .... S4
Figure S3 .... S5
Figure S4 .... S6
Figure S5 .... S7
Figure S6 .... S8
Figure S7 .... S9
Figure S8 .... S10
References S10

Table S1. Droplet sample generation parameters, including aqueous phase contents, oil phase, droplet volumes produced on PDMS, range for the number of samples produced per volume, analysis methods, and qualitative measure of GABA detection.

Sample ID	Aqueous/ Continuous Phase	Oil/Dispersed Phase	Droplet Volumes	Sample Count	Analysis Method	GABA Detection
A	100 mM GABA in aCSF	FC-40:PFO 10:1	300 pL – 1 nL	6-19	MALDI-MSI	Good
B	100 μM GABA in aCSF	FC-40:PFO 10:1	65 – 380 pL	4-11	MALDI-MSI	Good
C	100 μM GABA in aCSF	PFD:PFO 10:1	200 pL	5	MALDI-MSI	Poor
D	100 μM GABA in aCSF	PFO	200 pL	5	MALDI-MSI	Poor
E	100 μM GABA in aCSF	Octanol	65 pL	8	MALDI-MSI	Poor
F	100 μM GABA in aCSF	FC-40	65 pL	3	MALDI-MSI	Good
G	100 mM GABA in aCSF	FC-40	65 pL	2	MALDI-MSI	Good
H	100 μM Fluorescein and 100 μM GABA in aCSF	FC-40:PFO 10:1	500 nL & 65 pL	5	Optical Microscopy; MALDI-MSI of 65 pL	N/A; Good
I	100 mM GABA in aCSF	FC-40:PFO 10:1	65 pL	10	Raman; MALDI-MSI	Good; Good

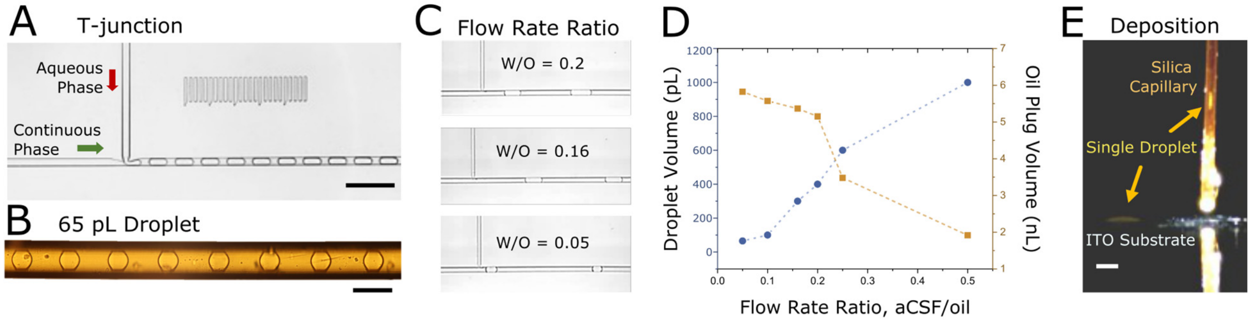

Figure S1. Droplet volume control and deposition on MALDI substrate. (A) PDMS-based T-junction microfluidic device for droplet generation. GABA containing aCSF is applied as the aqueous phase and oil is applied as the continuous phase. Scale bar, 200 μm. (B) 65 pL droplet sequence in silica capillary (I.D. 50 μm, O.D. 150 μm); the droplet shape is a 25 μm-radius sphere, droplet volume is 65 pL. Scale bar, 150 μm. (C) Optical images of droplet volumes depending on the flow rate ratio of aqueous and oil phases. (D) Droplet volume (blue) and oil plug volumes (orange) as a function of flow rate ratio. (E) Single droplet deposition on ITO glass substrate. Droplet frequency and deposition speed are adjusted by controlling the total flow rate. Scale bar, 200 μm.

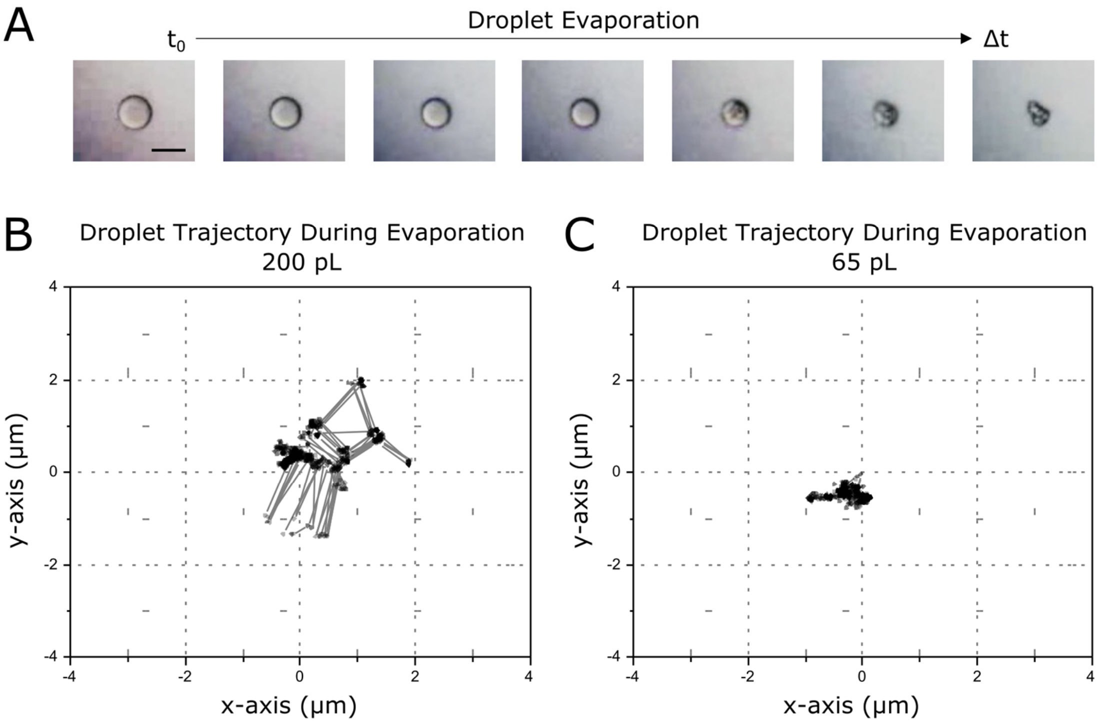

Figure S2. Trajectory analysis of evaporating droplet movement on ITO substrate using particle tracking method.[1] All droplets are produced in FC-40:PFO and contain 100 μM GABA. (A) Time-lapsed images of droplet evaporation process for Δt. Scale bar, 100 μm. (B) Trajectory of 200 pL aCSF droplet during evaporation. Maximum displacement of droplet trajectory is 2.52 μm and 3.42 μm in x- and y-axis, respectively. (C) Trajectory of 65 pL aCSF droplet during evaporation. Maximum displacement of droplet trajectory is 1.08 μm and 0.73 μm in x- and y-axis, respectively.

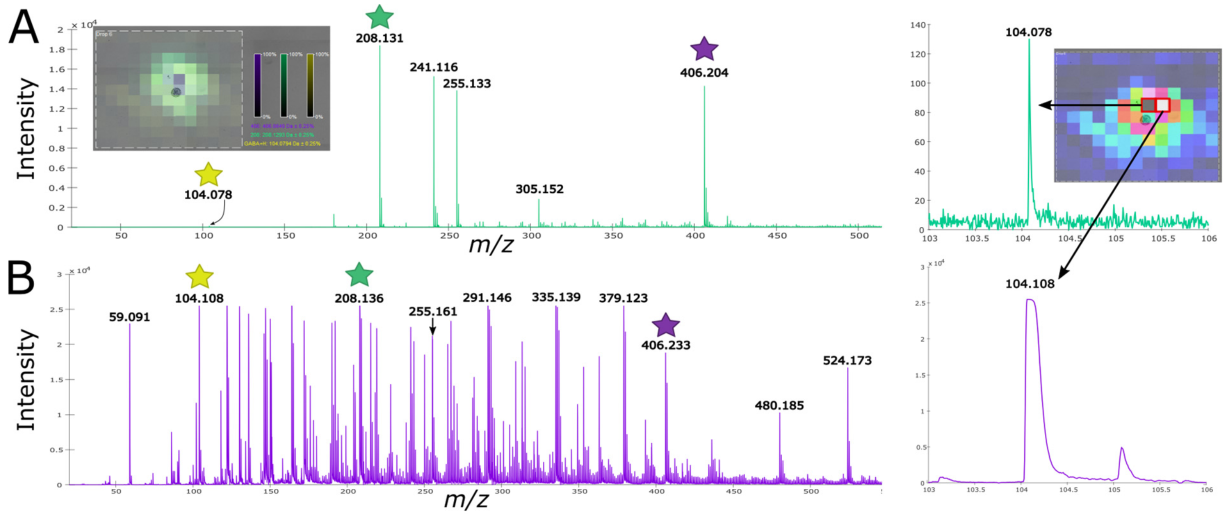

Figure S3. Representative mass spectra acquired at the (A) crystalline droplet sample core and (B) an adjacent pixel. Full mass spectra are shown on the left with zoomed spectrum for *m/z* region, including *m/z* 104 corresponding to $[GABA+H]^+$, on the right. Panel A: Insets are ion images illustrating the distribution of relevant ion species. Left inset: ion image shows overlaid signals for $[GABA + H]^+$ (yellow), *m/z* 208 (green), and *m/z* 406 (purple), these ions are not seen in the blank and colocalize with the droplet. Right inset: ion image shows rainbow scale for GABA signal intensities distribution where black/blue is low and pink/white is high intensity. Panel B, right: mass spectrum of GABA signal acquired at high laser intensity, leading to an overloaded first monoisotopic peak.

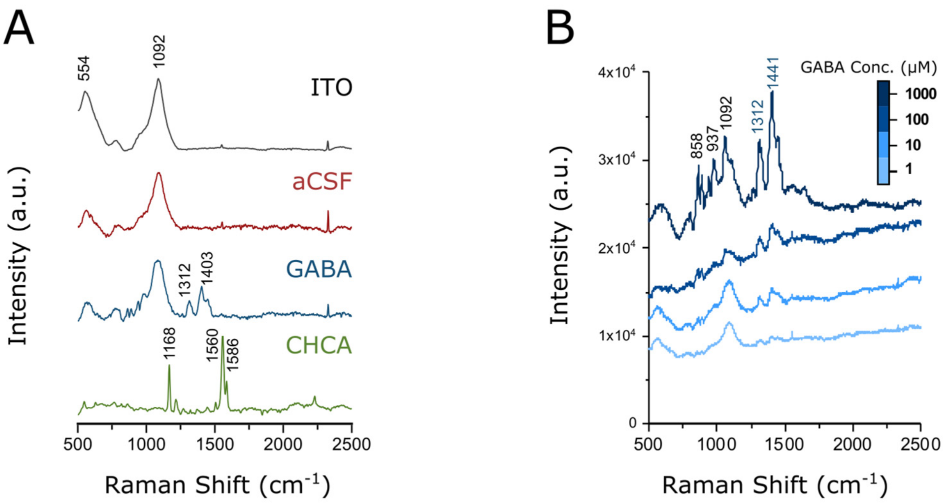

Figure S4. Characterization of Raman performance for MALDI-MSI samples. (A) Representative Raman spectra of (black line) ITO substrate (predominant peaks, 554, 1092 cm^{-1}); (red) aCSF; (blue) 100 μM GABA in 65 pL droplet (1312, 1403 cm^{-1}); (green) CHCA (1168, 1560, 1586 cm^{-1}) acquired at a wavelength of 532 nm. (B) Raman spectra of 1 to 1000 μM GABA deposited onto ITO glass slide in 1 μL aCSF droplets.

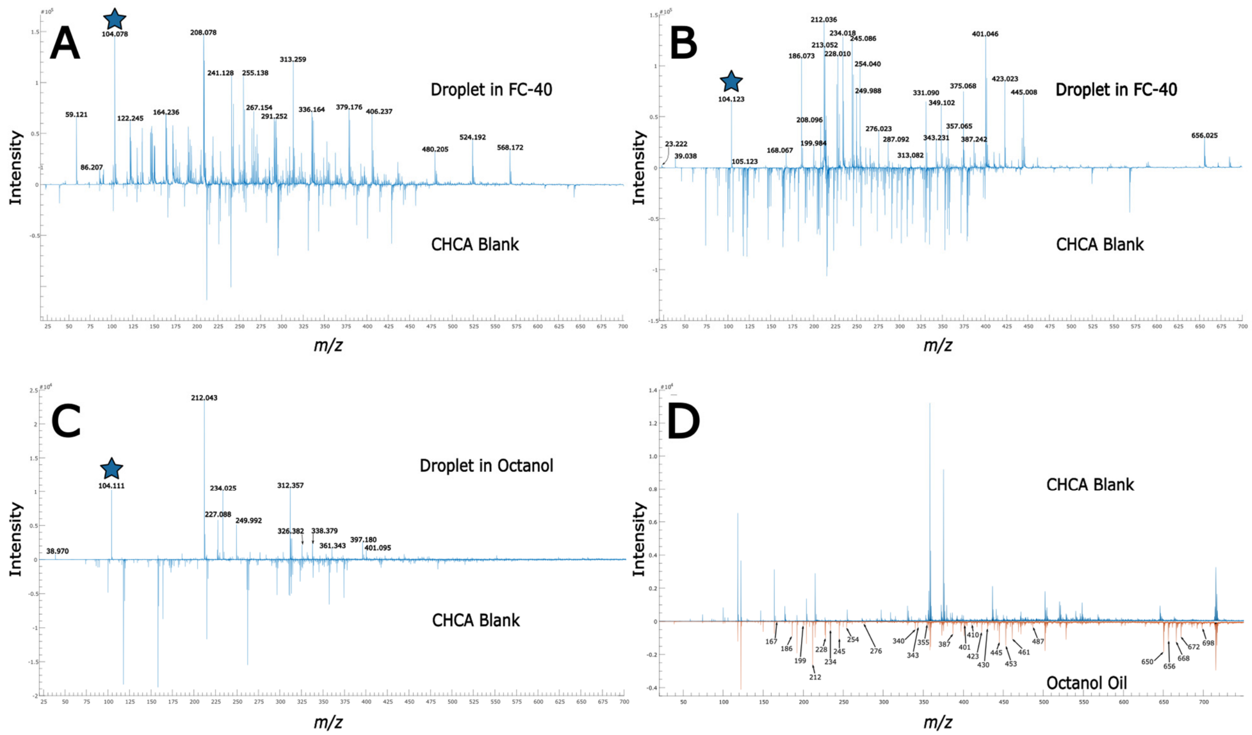

Figure S5. Representative subtracted mass spectra acquired from samples prepared using droplets produced in FC-40 and octanol. Spectra labeled by oil correspond to the average spectra for pixels with the highest intensity GABA signal within the droplet area. GABA signals are marked with a blue star in each spectrum. Blank spectra are acquired adjacent to the droplet to best capture the local environment without presence of GABA and therefore may contain oil phase. (A) 1 nL droplet produced in FC-40 with 10% v/v PFO containing 100 pmol of GABA. (B) 65 pL droplet produced in FC-40 with 10% v/v PFO containing 6.5 fmol of GABA. (C) 65 pL droplet produced in octanol containing 6.5 fmol of GABA. (D) Subtracted spectrum produced from an octanol oil plug between droplets in PDMS, deposited separately on ITO and a matrix blank.

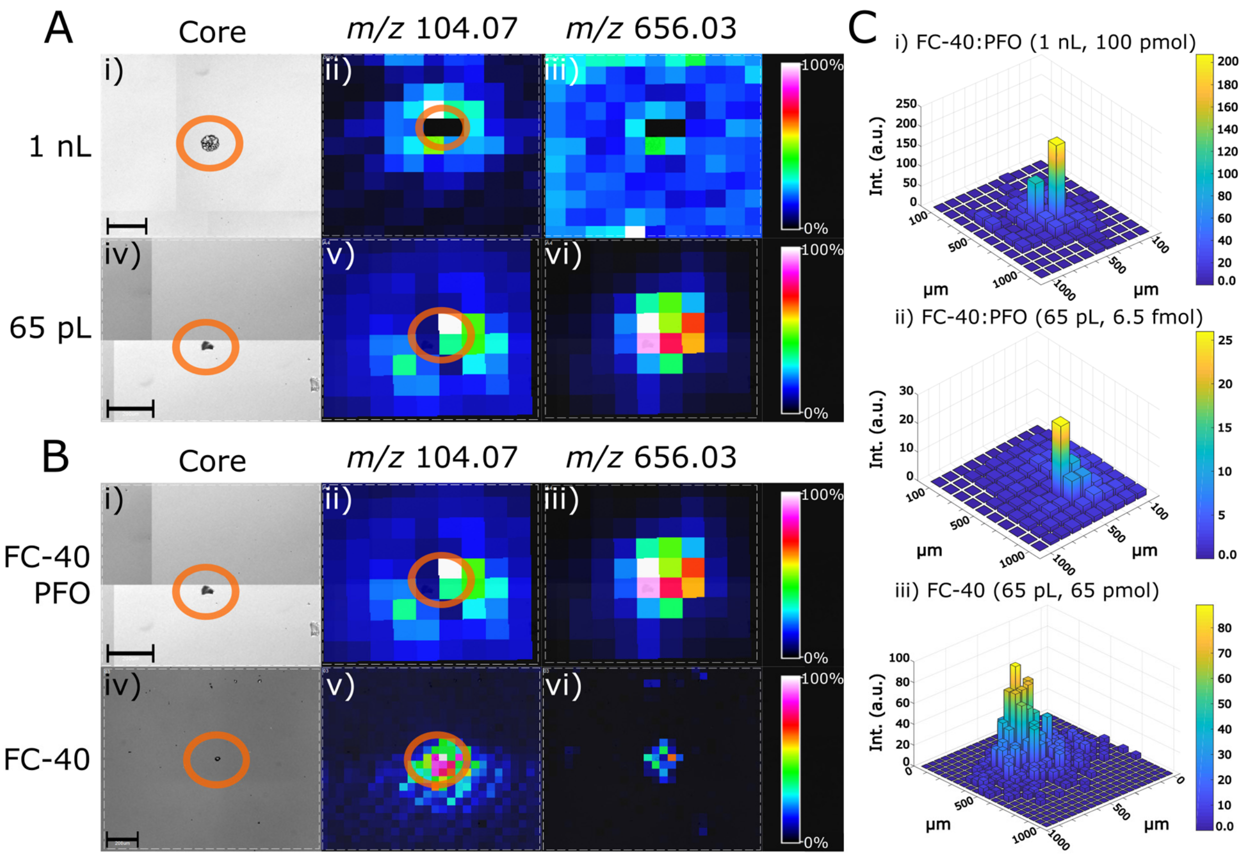

Figure S6. Comparison of MSI patterns of ion distribution across different droplet production parameters. Scale bars in A(i), A(iv), B(i), and B(iv) are 200 μm. All MS images were collected using 100 μm spatial resolution, except in the case of surfactant-free FC-40-mediated droplets, which were collected at 50 μm spatial resolution. (A) Comparison of droplet volumes (therefore moles of GABA). Both droplet volumes are produced in FC-40:PFO oil phase. Panel (i) shows the droplet core, (ii) MS image for *m/z* 104.07, (iii) MS image for *m/z* 656.03; repeated for (iv–vi). 1 nL droplets contain 100 pmol of GABA, 65 pL droplets contain 6.5 fmol of GABA. Delocalization of *m/z* 104.07 is similar across volumes, ~600 μm diameter, while *m/z* 656.03 is not as apparent in the 1 nL droplets with extensive salt crystallization. (B) Comparison of 65 pL droplets produced in FC-40 oil phase with or without surfactant PFO. Panel (i) shows the droplet core, (ii) MS image for *m/z* 104.07, (iii) MS image for *m/z* 656.03; repeated for (iv–vi). Delocalization of *m/z* 104.07 is similar across oil types, ~600 um diameter, whereas the extent of *m/z* 656.03 signal is 100 to 200 μm smaller. (C) Thresholded *m/z* 104.07 ion images for all droplet production parameters. The FWHM of the signal distribution in the x-y plane is 100 to 200 μm.

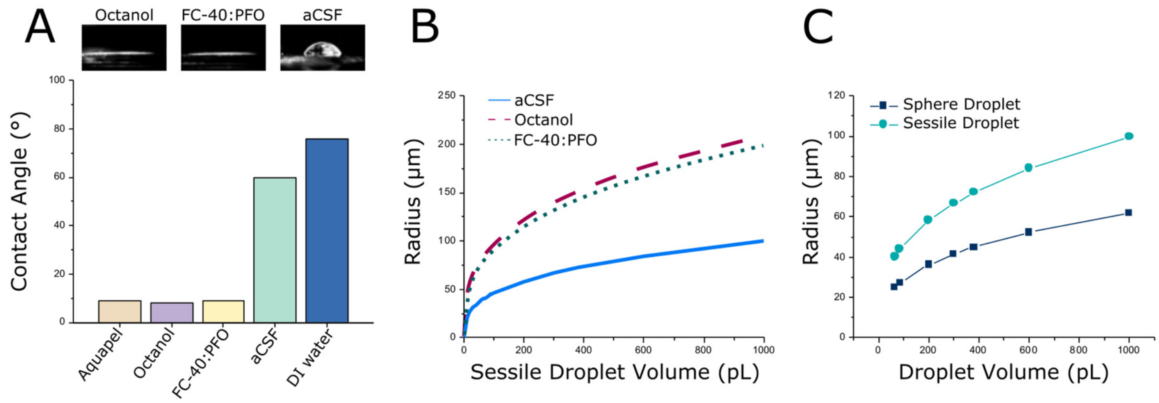

Figure S7. Calculation of sessile droplet areas deposited on ITO substrate. (A) Top: optical micrscopy images of different solution types. Bottom: Contact angle measurements of different type of solutions. The contact angles were measured using a Dino-Lite microscope in 0° projection and analyzed using ImageJ software. The ITO substrate has a hydrophobic property with an affinity for oils such as octanol and FC-40:PFO. (B) Theoretical contact area of sessile droplets. Assuming that the droplet is sufficiently small and gravity is negligible, sessile droplet volume, $\boldsymbol{V}$ is calculated using this formula[2], $\boldsymbol{V} = \frac{\pi R^3}{3}\frac{sin\theta(2+cos\theta)}{(1+cos\theta)^2}$ ($\boldsymbol{R}$: Contact radius, $\boldsymbol{\theta}$: Contact angle). (C) Contact areas of sphere shape droplet and sessile droplet of aCSF.

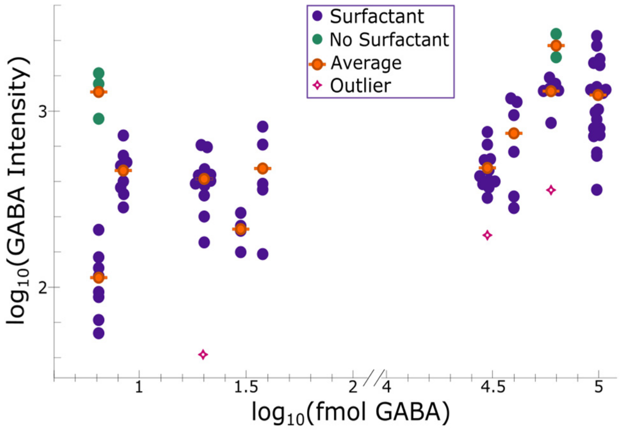

Figure S8. Comparison of total GABA intensity for droplets produced in surfactant-containing vs. surfactant-free oil phases. Two concentration regimes were used for droplets produced in FC-40:PFO (purple data points): 100 mM regime on the right hand side and 100 μM regime on the left hand side. Surfactant-free, 65 pL droplets were produced in both regimes (green data points). The surfactant-free droplets show a marked increase in GABA intensity, most prominent in the 100 μM regime with a ~10 fold increase.

References

(1) Park, I.; Lim, J. W.; Kim, S. H.; Choi, S.; Ko, K. H.; Son, M. G.; Chang, W.-J.; Yoon, Y. R.; Yang, S.; Key, J.; Kim, Y. S.; Eom, K.; Bashir, R.; Lee, S. Y.; Lee, S. W. Variable Membrane Dielectric Polarization Characteristic in Individual Live Cells. *J. Phys. Chem. Lett.* **2020**, *11* (17), 7197–7203. https://doi.org/10.1021/acs.jpclett.0c01427.

(2) Shin, D. H.; Lee, S. H.; Jung, J.-Y.; Yoo, J. Y. Evaporating Characteristics of Sessile Droplet on Hydrophobic and Hydrophilic Surfaces. *Microelectron. Eng.* **2009**, *86* (4), 1350–1353. https://doi.org/10.1016/j.mee.2009.01.026.
